# Health-Promoting Properties and Potential Application in the Food Industry of *Citrus medica* L. and *Citrus* × *clementina* Hort. Ex Tan. Essential Oils and Their Main Constituents

**DOI:** 10.3390/plants12050991

**Published:** 2023-02-21

**Authors:** Rosa Tundis, Jianbo Xiao, Ana Sanches Silva, Filipa Carreiró, Monica Rosa Loizzo

**Affiliations:** 1 Department of Pharmacy, Health and Nutritional Sciences, University of Calabria, 87036 Rende, Italy; 2 Nutrition and Bromatology Group, Analytical and Food Chemistry Department, Faculty of Food Science and Technology, Universidade de Vigo, Ourense Campus, E-32004 Ourense, Spain; 3 School of Food and Biological Engineering, Chengdu University, Chengdu 610106, China; 4 National Institute for Agrarian and Veterinary Research (INIAV), I.P., Rua dos Lágidos, Lugar da Madalena, Vairão, 4485-655 Vila do Conde, Portugal; 5 Faculty of Pharmacy, University of Coimbra, Polo III, Azinhaga de St. Comba, 3000-548 Coimbra, Portugal; 6 Centre for Animal Science Studies (CECA), ICETA, University of Porto, 4501-401 Porto, Portugal

**Keywords:** Rutaceae, peel, leaves, flowers, bioactivity, volatiles

## Abstract

*Citrus* is an important genus in the Rutaceae family, with high medicinal and economic value, and includes important crops such as lemons, orange, grapefruits, limes, etc. The *Citrus* species is rich sources of carbohydrates, vitamins, dietary fibre, and phytochemicals, mainly including limonoids, flavonoids, terpenes, and carotenoids. *Citrus* essential oils (EOs) consist of several biologically active compounds mainly belonging to the monoterpenes and sesquiterpenes classes. These compounds have demonstrated several health-promoting properties such as antimicrobial, antioxidant, anti-inflammatory, and anti-cancer properties. *Citrus* EOs are obtained mainly from peels, but also from leaves and flowers, and are widely used as flavouring ingredients in food, cosmetics, and pharmaceutical products. This review focused on the composition and biological properties of the EOs of *Citrus medica* L. and *Citrus clementina* Hort. Ex Tan and their main constituents, limonene, γ-terpinene, myrcene, linalool, and sabinene. The potential applications in the food industry have been also described. All the articles available in English or with an abstract in English were extracted from different databases such as PubMed, SciFinder, Google Scholar, Web of Science, Scopus, and Science Direct.

## 1. Introduction

Essential oils (EOs) are liquid mixtures of volatile molecules produced by aromatic plants. They have been used for millennia as antimicrobial, antioxidant, anti-inflammatory, relaxing, and stimulating substances, and isolated by a variety of procedures that have been refined over the centuries [1,2]. EOs are frequently used in the food and cosmetic industries for the production of food flavouring, shampoos, soaps, lotions, hair styling products, cologne, laundry detergents, and insect repellents.

The most abundant EO constituents are hydrocarbons, which are made up almost exclusively of terpenes (mainly monoterpenes and sesquiterpenes), and oxygenated compounds, which are mainly alcohols, aldehydes, ketones, esters, phenols, and oxides [3]. In general, EOs contain about 20–60 components and have up to 100–150 or more single molecules, at quite different concentrations [4]. In fact, generally, EOs have two or three major components at high concentrations and other compounds are present in small contents or traces [5]. Many factors, mainly including plant ecotype, genetic variation, plant nutrition, geographic location, seasonal variations, surrounding climate, possible stress during growth, post-harvest drying, and storage, affect the chemical profile of EOs [6].

EOs from the *Citrus* species are widely used as flavouring ingredients in food, cosmetics and pharmaceutical products [7].

*Citrus* is an important genus in the Rutaceae family with high medicinal and economic value [8]. There are 33 accepted species names for the *Citrus* genus. Different *Citrus* species are used in the food and cosmetic industries [9,10]. *Citrus* species are sources of carbohydrates, vitamins, dietary fibres, and several phytochemicals, mainly including limonoids, flavonoids, terpenes, and carotenoids, which have demonstrated a wide range of biological activities such as anti-inflammatory, immunomodulatory, antidiabetic, antioxidant, and neuroprotective activities [11,12,13,14].

*Citrus* EOs are obtained mainly from the fruit rind (flavedo), but also from leaves and flowers. The extraction is generally based on hydro-distillation, mainly in Clevenger-type apparatus, but they also can be obtained from peel by cold-pressing extraction [7]. Volatile and semi-volatile constituents represent about 85–99% of the oil fraction [15,16,17], usually consisting of over 200 compounds.

## 2. *Citrus medica* L. and *Citrus* × *clementina* Hort. Ex Tan.

The taxonomic classification of the *Citrus* species was complex and often controversial, and it has been subjected to intensive discussion [18,19]. Recently, despite the difficulty in establishing a consensual taxonomic classification of edible *Citrus* species, several authors reached agreement on the origin of cultivated *Citrus* species. The use of genome sequencing and different molecular markers have allowed them to identify four basic taxa, namely *C. medica*, *C. maxima*, *C. reticulata*, and *C. micrantha* as the origin of all edible *Citrus* species, and to decipher the origin of the other *Citrus* secondary species [18,20].

In this paper, we focused our attention on the EOs obtained from the *Citrus* species of great interest not only for the food industry, but also from a pharmaceutical and cosmetic point of view, such as *Citrus medica* L. and *Citrus clementina* Hort. Ex Tan.

*C. medica* L. (citron), native to southeast Asia, was the first *Citrus* fruit introduced to the Mediterranean area, and it was used for its aroma and as symbol in the Jewish religion. Citron remained the only citrus representative until the tenth century. Then, its cultivation was extended, reaching a peak in the nineteenth century, when it was also used in confectioneries and cosmetics [21]. The better-known *C. medica* varieties are Diamante, Corsican, Fingered citron, and Etrog [18,19,20,21].

*C. medica* cv. Diamante is cultivated in the stretch of the Tyrrhenian coast of Cosenza (Calabria, Southern Italy), also known as “Riviera dei Cedri”, between Tortora and Diamante. Here is where *C. medica* L. cv. Diamante finds its natural habitat, thanks to a microclimate characterized by mild temperatures all year round, without thermal excursions. In this area, 98% of the national production of *C. medica* L. cv. Diamante is obtained. *C. medica* L. cv. Diamante fruits are used in the food industry to prepare liqueurs and candy, while its essential oil is used as a flavouring in beverages and sweets [22].

*C. medica* L. cv. Corsican is the only one of the *C. medica* group that has sweet albedo and acid-free pulp. The fruit skin is thick, and its albedo is consumed usually after being processed into jam or candied fruit [23]. In Corsica, the main product obtained from the peel is a liqueur called “Aliméa” in the Corsican language or “Cédratine” in French.

*Citrus medica* L. var. *sarcodactylis* (fingered citron, also known as nine-claw wood, citrus chirocarpus, or five-fingered orange) has its origins in India, but it soon spread all over the world. Now, it is mainly present in Guangxi, Zhejiang, Sichuan and other parts of China. It is a shrub or small tree. The stem has short spines and its leaves are alternate. During the fruit development, the fingered citron is divided into finger-shaped meat strips. Its peel, leaves, and root have been used in traditional medicine for the treatment of infectious hepatitis, oedema, headache, rheumatism, and arthritis [24].

*Citrus medica* L. var. *Etrog* comes from small and shrubby trees with oblong but slightly pointed leaves that are somewhat rumpled with serrate margins [25]. The mature fruits, very fragrant with a distinctive aroma, are considerably larger than lemons, with a glossy, yellow, thick, and bumpy rind. The pulp is pale yellow and acidic, but not very juicy. The seeds are numerous. Fruits of this variety are mainly used for Jewish ritual in the Sukkot harvest festival.

*Citrus* × *clementina* fruits (clementine) grow on different continents. In Europe, Italy is among the major producers. In particular, in Calabria, the cultivation of clementine is widespread due to the optimal climatic conditions, which contributed to the development of products awarded with Protected Geographical Indications certification in 1997 by the European Commission as “Clementine di Calabria IGP” [26]. *Citrus* × *clementina* is a *Citrus* fruit hybrid of mandarin and sweet orange. The clementine tree is very similar to the mandarin tree, though it has leaves that are larger and wider. *C.* × *clementina* fruits are usually seedless and typically juicy and sweet, with less acid than oranges [22].

## 3. Methodology

An extensive survey of the chemical constituents and biological properties of *C. medica* and *C.* × *clementina* EOs was conducted in scientific databases PubMed, SciFinder, Google Scholar, Web of Science, Scopus, and Science Direct. The search terms “*Citrus* genus”, “*Citrus* × *clementina*”, “*Citrus medica*”, “*Citrus medica* essential oil”, “*Citrus* × *clementina* essential oil”, “*Citrus medica* biological activity”, “*Citrus* × *clementina* biological activity”, “antioxidant”, “antimicrobial”, “anti-inflammatory”, “*Citrus medica* enzyme inhibition”, “*Citrus* × *clementina* enzyme inhibition”, “application in food”, and “toxicity” were used for data collection. The inclusion criteria included research articles and reviews that reported the chemical profile and the bioactivity of *Citrus medica* and *Citrus* × *clementina* EOs; articles published in English; and all studies that evidenced cellular and molecular mechanisms of action in cell cultures, animal models and preclinical and clinical models. Articles focused on extracts and non-volatile compounds, and their biological activities were excluded.

## 4. Chemical Composition

Many chemical constituents have been identified in the EOs from *Citrus medica* and *C.* × *clementina,* categorized into monoterpene hydrocarbons, oxygenated monoterpenes, sesquiterpene hydrocarbons, oxygenated sesquiterpenes, diterpenes, oxygenated diterpenes, acyclic diterpenoids, aliphatic alkanes and alkenes, aliphatic alcohols, aldehydes, ketones, esters, fatty acids, and other components. The most used parts to obtain EOs are the peels, followed by the leaves. Concerning the extraction method, hydrodistillation was the major method of choice, while some studies also used steam distillation, peel cold pressure, and supercritical fluid extraction.

### 4.1. C. medica L.

Due to the low ratio of EO:fruit weight, *C. medica* EO is a minor product, but it is used not only as aromatic ingredient in confectionery, cosmetics, and beverages, but also for its healthy properties, which were already recognized by the ancient Greeks and Romans. We reported here on the analysis of the chemical composition of *C. medica* EO from the peel and leaves.

**Figure 1:**
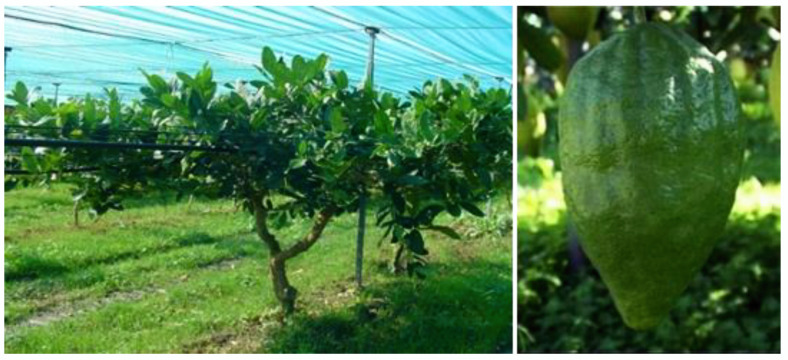
*Citrus medica* cv. Diamante.

*C. medica* L. cv. Diamante (Figure 1) EO obtained by hydrodistillation is characterised by the presence of monoterpene hydrocarbons as major components.

Table 1 reports the chemical profile of *C. medica* L. cv. Diamante from Italy [27,28,29]. The most abundant compounds of the EO from Calabria (Southern Italy) are limonene (35.4%) and γ-terpinene (24.5%) [27]. This is in agreement with the results obtained by Poiana et al. [28] and Gabriele et al. [29] that evidenced that these constituents are characteristic of this *Citrus* cultivar. However, the analysed EOs evidenced some differences in the content of other volatiles, mainly geranial and neral. Moreover, citropten (0.9%) was identified only in the EO obtained by Menichini et al. [27].

Germacrene B, (E)-nerolidol, and spathulenol have been found in trace only by Poiana et al. [28].

The EO from the peel and leaves of *C. medica* cv. Diamante from Crete (Greece) showed limonene as the main component, with values of 249.3 and 205.4 mg/kg for peel and leaves, respectively [30]. Other compounds found with a high content in the peel EO are geranial (133.7 mg/kg) and neral (80.8 mg/kg), followed by geraniol (22.3 mg/kg), nerol + citronellol (20.5 mg/kg), myrcene (12.59 mg/kg), β-pinene (12.54 mg/kg), neryl acetate (10.57 mg/kg), geranyl acetate (6.32 mg/kg) and linalool (5.31 mg/kg).

These constituents also characterized the EO from leaves. However, some differences can be highlighted. Leaves EOs were richer in neral (165.2 mg/kg), geranial (175.8 mg/kg), and geranyl acetate (38.5 mg/kg) than peel EOs. On the other hand, peel EO has a higher content of nerol + citronellol and geraniol.

Aliberti et al. [31] analysed the chemical profile of *C. medica* cv. “liscia” and *C. medica* cv. “rugosa,” two citron taxa from Amalfi Coast, Campania region (Italy).

Monoterpene hydrocarbons are the main constituents in both EOs (79.1 and 80.2% for *C. medica* cv. “liscia” and “rugosa”, respectively). In both EOs, limonene (67.2–62.8%) and camphene (8.5–10.9%) are the dominant constituents, followed by β-pinene (1.4–1.7%) and α-pinene (0.8–1.2%).

Two chemotypes of *C. medica* var. *sarcodactylis* EO from Vietnam were found, one that showed limonene and *p*-cymene and the other that principally contained limonene and γ-terpinene [32]. Previously, the chemical profile of the peel EO from Japanese *C. medica* var. *sarcodactylis* was analysed [33]. In this case, the main components were limonene (47.8%), and γ-terpinene (32.1%), followed by α-pinene (2.9%), geranial (2.5%), myrcene (1.7%), neral (1.6%), terpinolene (1.4%) and γ-thujene (1.33%).

In agreement with these data are the results obtained by Venturini et al. [18], who studied the peel EO of *C. medica* var. *sarcodactylis* from Spain, finding limonene (39.5%) and γ-terpinene (28.6%) as the most abundant compounds, followed by geranial (5.2%), *p*-cymene (3.4%), neral (3.6%), α-pinene (3.2), and myrcene (1.7%). Limonene (41.8%), geranial (17.9%), neral (13.6%), nerol (4.1%), and linalool (1.0%) are the dominant constituents of the *C. medica* var. *sarcodactylis* EO from leaves.

*C. medica* cv. Corsican EO composition, according to the development stage of the fruit, was analysed by Venturini et al. [23], who reported 20 monoterpene hydrocarbons, 20 oxygenated monoterpenes, 4 sesquiterpene hydrocarbons, and 2 linear oxygenated components as major constituents. Monoterpene hydrocarbons (66.8–82.5%) represent the main class of compounds. Limonene was the major compound (54.2–60.6%), followed by γ-terpinene (6.7–15.2%), nerol (2.9–8.2%), neral (2.1–6.6%), geraniol (1.8–5.9%), and geranial (2.1–5.7%). Two different chemical compositions of Etrog peel EOs were identified: limonene/γ-terpinene or limonene/neral/geranial. The first chemical composition is in agreement with that reported for EOs from Corsica [34], while the second composition is in accordance with that reported for EOs from Italy and Israel [35,36,37].

### 4.2. C. × clementina Hort. Ex Tan.

**Figure 2:**
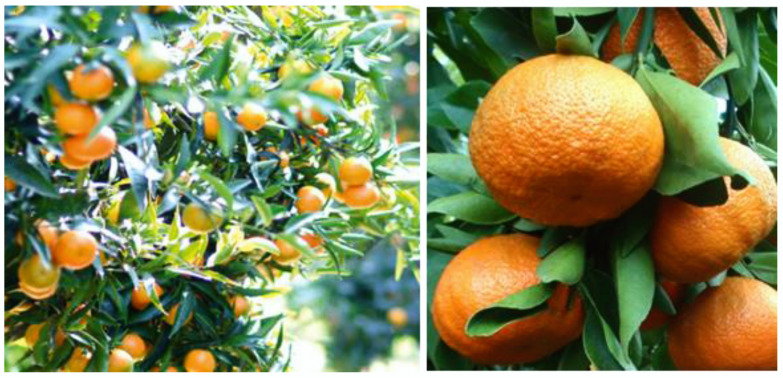
*Citrus* × *clementina* Hort. Ex Tan.

*C.* × *clementina* fruits (Figure 2) are hybrids between *C. sinensis* and *C. reticulata*, and are cultivated throughout the world, especially in the Mediterranean region and Asia. In Europe, Italy is the major producer. In Vietnam, *C.* × *clementina* fruits are considered one of the main consumed *Citrus* fruits.

Kirbalar et al. [38] compared the clementine peel extract produced from Turkish-grown clementines (*C.* × *clementina* Hort. Ex Tan.) using two methods: cold pressing (CP) and supercritical CO_2_ extraction (SFE) (Table 2).

Gas chromatography (GC) and gas chromatography/mass spectrometry (GC/MS) were used to evaluate the chemical contents of the volatile extract samples. Sixty-nine components were found, accounting for 99.8% of the total volatiles in both samples. Compared to the SFE extraction, the CP extraction produced less oxygenated compounds (3.7%); the majority of them were carbonyls (2.09–2.10%), followed by alcohols (1.32–1.60%) and esters (0.12–0.40%). Myrcene (4.64–3.77%) came in second place to limonene (88.12–89.28%) as the primary constituent [38].

Linalool (1.02–1.24%) and decanal (0.71–0.72%) were found to be the most abundant oxygenated substances. The method used to obtain the extract had influence in the final composition. For instance, linalyl acetate was determined in the CP extract at trace levels but it was present at 0.14% in the extract obtained by SFE.

Thi Nguyen et al. [39] compared the composition of essential oils extracted from the peels and leaves of *C. clementina* Hort. ex Tan. using two other methods: conventional hydrodistillation (CHD) and microwave-assisted hydrodistillation (MAHD). In comparison to CHD, the microwave irradiation approach was able to provide a more efficient extraction (greater oil yield and higher percentage of oxygenated components). A total of 41 and 67 compounds of the total essential oil composition were identified in peels and leaves, respectively. The major compounds in peel EOs were monoterpene hydrocarbons, with limonene being the most relevant (Table 2).

Sesquiterpene hydrocarbons were the predominant constituents of the EOs obtained from the leaves, with sabinene, β-elemene, linalool, (E)-β-ocimene, β-caryophyllene, and δ-selinene as the main components (Table 3) [39].

Ruberto et al. [42] studied the profile of two hybrids of *C. clementina* × *C. sinensis* (A146 and C1867) and their parents and grouped the 42 identified compounds into monoterpene hydrocarbons, oxygenated monoterpenes, sesquiterpenes and aliphatic aldehydes/alcohols.

Limonene was found to be the most abundant compound, corresponding to 95% of the total oils. β-Pinene, α-terpinene, β-phellandrene, and γ-terpinene were present in slightly higher amounts in both hybrids, while α-pinene, myrcene and 3-carene were found in higher amounts for the C1867 hybrid.

Miguel et al. [43] examined the EOs from *C. sinensis* (L.) Osbeck (Cs) and *Citrus clementina* Hort. Ex Tan. flowers (Table 4). According to their results, a yellow oil was produced with yields between 0.05 and 0.08% (*v*/*w*). Both EOs were dominated by the monoterpene fraction (66–91%), with monoterpene hydrocarbons making up the majority of this fraction (45–69%). Sabinene (31–48%), linalool (15–32%), and limonene (4–10%) were the most abundant constituents. With a percentage of 3–10%, (E)-nerolidol was the main identified sesquiterpene [43].

In order to learn more about “gametoclonal variation”, or the variation produced by the gametic embryogenesis process, Germaná et al. [44] compared the composition of the essential oils obtained by the Clevenger apparatus from the leaves of pollen-derived homozygous plants with those obtained from the heterozygous clementine and from its ancestors, sweet orange and mandarin [44].

Principal components analysis (PCA) was used to divide the resulting oil compositions into groups and distinguish them from those of both parents in terms of three key chemotypes: sabinene/linalool, γ-terpinene, and methyl *N*-methylanthranilate.

Due to its chemical composition, the essential oil of *C.* × *clementina* Hort. Ex Tan. has great potential for use by different industries, such as the industries of perfumes, cosmetics, food and pharmaceuticals.

## 5. Biological Properties

Based on their properties, *Citrus* EOs are widely used in the food, cosmetic, and pharmaceutical industries [7]. Among the most used and studied *Citrus* species are *C. limon*, *C. aurantifolia*, *C. aurantium*, *C. sinensis*, *C. bergamia*. Recently, other species such as *C. medica* and *C.* × *clementina* have attracted the interest of researchers. The antimicrobial, antioxidant, anti-inflammatory, neuroprotective, and enzyme-inhibitory activities have been investigated.

### 5.1. Antibacterial and Antifungal Properties

The EO obtained from the peel of *Citrus medica* cv. ‘liscia’ and *C. medica* cv. ‘rugosa’, two cultivars of *C. medica* growing on the Amalfi Coast (Southern Italy), were studied for their antibacterial activity [31]. Generally, EO from *C. medica* cv. ‘liscia’ exhibited a greater inhibitory activity against the growth of all tested bacteria strains (*Bacillus cereus* DSM 4313 and DSM 4384, *Escherichia coli* DSM 8579, *Pseudomonas aeruginosa* ATCC 50071, and *Staphylococcus aureus* DSM 25693), with Minimum Inhibitory Concentration (MIC) values of 0.1 μL/mL, and was much more effective than the positive control gentamicin. *P. aeruginosa* and *E. coli* were the most sensitive strains to EO obtained from *C. medica* cv. ‘liscia,’ with a diameter of inhibition of less of 27 mm, whereas a diameter superior to 22 mm was never observed with the *C. medica* cv. ‘rugosa’ EO against *S. aureus* [31].

The antibacterial activity of *C. medica* was confirmed also by the study of Mitropoulou et al. [45]. The peel EO was tested against *Aspergillus niger*, *Escherichia coli*, *Listeria monocytogenes*, *Saccharomyces cerevisiae*, *Salmonella enteritidis*, *Salmonella typhimurium*, *Staphylococcus aureus*, *Staphylococcus epidermidis* and *Pseudomonas fragi*. A large inhibition zone (35 mm after 1 day of incubation) was observed in *A. niger*. The MIC values for the EO ranged from 845 to 2008 mg/L for *S. epidermidis* and *P. fragi*, respectively. The Minimum Bactericidal Concentration (MBC) values were in the range 4184–8368 mg/L for *S. enteritidis* and *P. fragi*, respectively.

Guo et al. [46] demonstrated the anti-listeria activity of Chinese *C. medica* var. *sarcodactylus*. After EO treatment, the microorganism responded and adapted by increasing motility through the enhancement of flagella rotation; by promoting cell tumbles and re-orientating to escape from fingered *Citron* essential oil; by enhancing the uptake of carbohydrates from environment to gain more energy; and by changing the uptake of several metallic cations, including iron, zinc, cobalt, and nickel.

*C. medica* var. *sarcodactylis* peel EO exhibited antibacterial activity against different isolates. The following rank of inhibitory activity was found after 100 mL of EO application: *Staphylococcus aureus* (25 mm) > *Klebsiella pneumoniae* (22 mm) > *Proteus mirabilis*, *Bacillus* sp. and *Streptococcus* spp. (20 mm) > *Enterobacter cloacae* (18 mm), *Escherichia coli*, and *Pseudomonas aeruginosa* (15 mm) [47]. Generally, *C. medica* EO exerts a more pronounced action against Gram-positive bacteria than Gram-negative bacteria. This is probably related to the hydrophilic nature of the Gram-negative bacteria cell wall [48].

Conversely, EOs can directly damage the cell membrane of Gram-positive bacteria, with listeria breakdown of the cell membrane, blocking of enzymatic systems, and the progressivity of ionic permeability [49]. A diameter of inhibition of 9.15 mm, 11.15 mm and 8.02 mm was found for *Staphylococcus aureus*, *Propionibacterium acne*, and *Candida albicans* after listeria addition of 20 mL to listeria agar surface [50].

Li et al. [51] investigated the effect of *C. medica* var. *sarcodactylus* EO obtained from fruits collected in China. Results exhibited moderately antibacterial activity against *E. coli* (inhibition zone diameter 11.2 mm), *S. aureus* (inhibition zone diameter 19.2 mm), *B. subtilis* (inhibition zone diameter 16.3 mm) and *M. luteus* (inhibition zone diameter 16.1 mm), with a greater sensitivity of Gram-positive microorganisms than Gram-negative microorganisms.

Changes of *E. coli* and *S. aureus* bacteria morphology and the loss of permeability and integrity of the cell membrane was observed in a concentration-dependent manner. More recently, Vitalini et al. [52] explored the antibacterial effect of *C. medica* var. *sarcodactylus* EO and hydrolate from exocarp as well as methanol extracts from both exocarp and mesocarp (EEX and MEX). EEX showed higher inhibitory activity than MEX, with MIC and MBC values of 2.5 and 5 mg/mL, respectively, against *B. cereus*. A MIC value of 0.5% and MBC value of 1.0% against *B. cereus* were recorded for EO. On the contrary, the hydrolate did not exert antibacterial activity. The anti-leishmanial activity of EOs from the leaves of different *Citrus* species from Tunisia, including *C. clementina* EO obtained by a solvent-free microwave extraction procedure, was assessed [53]. This EO exerted the highest inhibitory activity, with IC_50_ values of 1.03 and 0.32 mg/mL against *Leishmania major* and *Leishmania infantum*, respectively. This effect could be attributed to the main abundant terpene limonene as well as the synergistic effect of the phytocomplex.

The antimicrobial activity (by disc diffusion antibiotic sensitivity assay) of the EOs extracted from peels and leaves of *C.* × *clementina* from Vietnam using hydrodistillation and microwave-assisted hydrodistillation against two Gram (+) bacteria (*B. subtilis* and *S. aureus*), three Gram (-) bacteria (*E. coli*, *P. aeruginosa*, and *Shigella flexneri*), and one fungus (*C. albicans*) was studied by Thi Nguyen et al. [39]. Peel EOs exhibited moderate activity against *B. subtilis* and *C. albicans*. Against the other bacteria, only the peel EO extracted by microwave-assisted hydrodistillation showed notable activity against *E. coli*. This activity may be related to the presence in this EO of a higher amount of oxygenated monoterpenes and thymol. The EOs from leaves revealed a strong inhibition against *S. aureus,* probably in relation to the abundance of oxygenated compounds.

**Figure 3:**
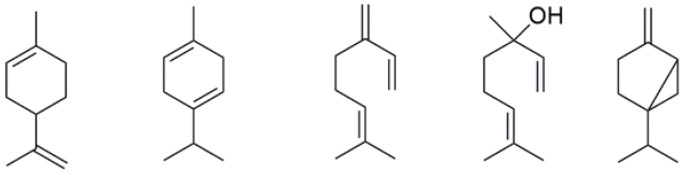
The main abundant constituents of *C. medica* and *C.* × *clementina* EOs.

Several studies analysed the antimicrobial potential of the dominant compounds of the EO of *C. medica* and *C.* × *clementina* (Figure 3). In particular, limonene has demonstrated its ability to inhibit Gram-positive and Gram-negative bacteria as well as fungal activities [54]. Furthermore, many works have confirmed that limonene can inhibit the growth of spoilage bacteria, such as *E. coli*, *Pseudomonas aeruginosa, Aspergillus niger*, and *S. aureus* [55,56].

1,8-Cineole, commonly known as eucalyptol, is abundant in various EOs (such as in eucalyptus and rosemary EOs), and due to its anti-inflammatory effects, it is used for the treatment of bronchial asthma, sinusitis, and chronic obstructive pulmonary disease [57,58].

However, several studies also reported its antibacterial activity against human pathogenic bacteria strains, specifically *B. subtilis*, *E. coli* O157:H7, *S. typhimurium*, *P. mirabilis*, *P. aeruginosa*, *S. aureus*, *S. epidermidis*, *S. enteritidis*, *Micrococcus favus*, and *Enterobacter cloacae* [57,59,60,61]. Against *E. coli* O157:H7, linalool was also proven to be active [62]. In addition to their lipophilicity, the described effects of monoterpenes are most likely due to the alteration of the bacterial membrane permeability and the increased membrane fluidity that causes changes in the topology of membrane proteins, giving rise to the interruption in the respiratory process [62,63].

### 5.2. Anti-Inflammatory Activity

Inflammation is a defense mechanism against infection and tissue damage that, if not stopped in time, can be the contributing cause of different pathologies such as cardiovascular and neurodegenerative diseases, diabetes and cancer [64]. Steroidal and nonsteroidal anti-inflammatory agents are commonly used for the treatment of inflammatory diseases. However, the long-term use of these drugs can cause serious side effects in the gastrointestinal tract, cardiovascular system, and liver. Finding a safe and effective anti-inflammatory drug has always been a challenge.

In recent years, EOs, and in particular *Citrus* EOs, have been rated as safe and promising anti-inflammatory drug candidates. Kim et al. [65] analysed the anti-inflammatory effects on lipopolysaccharide (LPS)-stimulated RAW 264.7 cells of *C. medica* var. *sarcodactylus* peel EO. This essential oil considerably inhibited the production of nitric oxide (NO) by suppressing the protein expression of inducible nitric oxide synthase (*i*NOS) and prostaglandin E2 by inhibition of the enzyme cyclooxygenase (COX)-2. Moreover, the authors demonstrated the ability of the EO to attenuate LPS-induced nuclear factor-κB (NF-κB) activation, to block the activation of extracellular signal-regulated kinase (ERK) and c-Jun *N*-terminal kinase (JNK), and to suppress tumor necrosis factor-α (TNF-α), interleukin (IL)-1β, and IL-6 production.

The anti-inflammatory activity of *C. medica* cv. Diamante peel EOs were studied by evaluating their inhibitory properties of NO production in LPS-stimulated macrophages [27]. EO obtained by hydrodistillation exerted promising activity, with an IC_50_ value of 17.0 μg/mL, in comparison to the positive control, indomethacin, which showed an IC_50_ value of 53.0 μg/mL. The EO obtained by cold-pressing extraction showed lower activity with an IC_50_ of 103.0 μg/mL, while the EO obtained by supercritical fluid extraction was not active.

The anti-inflammatory properties of the most abundant *C. medica* and *C.* × *clementina* EOs constituents (Figure 3), such as limonene [65,66], γ-terpinene [65], sabinene, 1,8-cineole [67], myrcene [68], and linalool [69,70], have been the object of different studies.

In LPS-activated RAW 264.7 cells, limonene reduced the production of NO, prostaglandin E2, TNF-α, IL-1β, and IL-6 in a concentration-dependent manner [66]. Kim et al. [65] investigated the inhibitory activity of prostaglandin E2 and the NO production of limonene and γ-terpinene. γ-terpinene exhibited weak activity, while limonene inhibited prostaglandin E2 production and suppressed LPS-induced NO production. Previously, another study has shown the inhibitory activity of the LPS-induced production of NO, PGE2, and pro-inflammatory cytokines in RAW 264.7 cells by limonene [66]. The effects of γ-terpinene have been evaluated by de Oliveira Ramalho et al. using several in vivo experimental models of acute inflammation [71].

γ-terpinene was able to reduce the production of TNF-α and IL-1β as well as neutrophil migration in a carrageenan-induced peritonitis model when compared to non-treated animals. Moreover, the pre-treatment of mice with γ-terpinene reduced paw oedema induced by carrageenan. Similarly, a reduction of paw oedema was observed when γ-terpinene was administered to the mice after stimulation with bradykinin, histamine, and PGE2. In the acetic acid model of microvascular permeability, treatment with the monoterpene inhibited fluid extravasation.

1,8-Cineole is one of the most investigated constituents of *Citrus* EOs as a potential therapeutic agent for the control and protection of inflammatory airway diseases [67]. This monoterpene inhibited the production of oxygen radicals, various cytokines and other inflammatory mediators. These effects have been confirmed in patients affected by asthma, chronic obstructive pulmonary disease (COPD), bronchitis, and sinusitis. 1,8-Cineole has been shown to be an efficient agent in co-medication for improving the standard treatments for COPD, asthma, and other inflammatory airway diseases.

In vitro β-myrcene has been shown to be a promising anti-inflammatory agent [72]. Its capacity to reduce inflammation occurs through PGE-2 [73]. In mouse models of pleurisy, myrcene was effective in inhibiting the inflammatory response induced by lipopolysaccharide, including cell migration and production of NO and the significant inhibition of γ-interferon and IL-4 [74]. β-Myrcene was involved in suppressing matrix metalloproteinases MMP-2 and MMP-9 and regulating the expression of iNOS [75]. In conclusion, the anti-inflammatory properties of β-myrcene may be ascribed to its interaction with several pathway cascades involving transcription factors and cytokines.

The anti-inflammatory effects of (±) linalool and (–) linalool have been studied in rats by using carrageenin-induced oedema as a model [76]. Both the pure enantiomer and its racemate produced, after systemic administration, a reduction in oedema. While the racemate showed a significant reduction of the oedema only 1 h after carrageenin administration, the effect of the pure enantiomer (at the dose of 25 mg/kg) was delayed and more prolonged. At higher tested doses, no differences were observed.

### 5.3. Antioxidant and Neuroprotective Effects

Neurodegenerative diseases (NDs) are a group of disorders that are characterized by cognitive or functional deterioration of neurons and share some processes associated with progressive neuronal dysfunction and death, including abnormal protein deposition, oxidative stress, neuro-inflammation, damaged mitochondrial function, and induction of apoptosis [77,78].

Several natural compounds have shown to be potential protective agents against oxidative stress and neuro-inflammation [79]. Moreover, a great number of phytochemicals have been described as promising acetylcholinesterase and butyrylcholinesterase inhibitory agents [80].

Alzheimer’s disease (AD) is one of the most common neurodegenerative diseases, accounting for 60–80% of all cases, and is characterized by cognitive decline and memory impairment [81]. In patients affected by AD, acetylcholine, the neurotransmitter essential for processing memory and learning, is decreased in both concentration and function. The treatment of AD can be divided into two main categories: symptomatic and disease-modifying. The disease-modifying treatments include amyloid binders, tau therapies, and secretase inhibitors. The symptomatic treatments include the use of antagonists of *N*-methyl-D-aspartate receptor and inhibitors of the enzyme acetylcholinesterase (AChE) responsible for the hydrolysis of acetylcholine in several cholinergic pathways. Like AChE, butyrylcholinesterase (BChE) also inactivates acetylcholine [80]. These enzymes differ in substrate specificity, kinetics, and activity in different brain regions, but are a useful target for improving the cholinergic deficit that characterizes AD. During recent decades, a number of new AChE and BChE inhibitors have been isolated from medicinal plants [80].

In this, context, Menichini et al. [27] analysed *C. medica* cv. Diamante EOs obtained by hydrodistillation, supercritical CO_2_ extraction, and cold-pressing extraction as potential inhibitory agents of AChE and BChE enzymes. In this work, EO obtained by hydrodistillation inhibited both AChE and BChE (IC_50_ of 171.3 and 154.6 μg/mL, respectively), EO obtained by cold-pressing showed a selective AChE inhibition (IC_50_ of 298.8 μg/mL), while EO obtained by supercritical CO_2_ extraction was inactive against both enzymes. Comparing the three EOs from the point of view of chemical composition, we can speculate on the role of some constituents. The AChE-inhibitory activity of some constituents identified in the *Citrus* EOs was previously described [82,83,84]. γ-terpinene, one of the most abundant compounds of *C. medica* cv Diamante EO, exhibited an IC_50_ value of 1 mM against AChE. Limonene was less active, with a percentage of inhibition of 27% at the concentration of 164.0 mg/mL [82].

Adenylyl cyclases (ADCYs), by generating second messenger cAMP, play important roles in various processes, including the regulation of multiple brain processes such as memory and synaptic plasticity. In this perspective, EOs of *C. medica* cv. liscia and *C. medica* cv. rugosa and their main constituent limonene were studied in order to evaluate their influence on the expression of ADCY1 [31]. A human-derived neuroblastoma cell line often used as a neuronal model, such as the SH-SY5Y cell line, was used.

The treatment of the SH-SY5Y neuroblastoma cell line with *C. medica* cv. liscia and *C. medica* cv. rugosa EOs at the concentrations of 400, 200, 100, and 50 μg/mL significantly influenced the expression of ADCY1 with an over-expression and a down-expression, respectively. Treatments with limonene significantly influenced ADCY1 expression. High concentrations increased ADCY1 expression, while low concentrations reduced ADCY1 expression. Limonene showed several biological properties such as anti-inflammatory, antioxidant, anti-cancer, and anti-nociceptive activities. Several studies also demonstrated the promising neuroprotective role of limonene in neurodegenerative diseases.

Moreover, the abundance of limonene in different plant species, its safety profile, and its mechanisms of action make this compound a favourable agent for treating neurodegenerative diseases [85]. The different results obtained by *C. medica* EOs are probably related to the different chemical composition. EOs and limonene were not cytotoxic for the SH-SY5Y cell line (IC_50_ values > 700 μg/mL).

As already reported, NDs in their pathogenesis are often characterised by not only abnormal protein deposition, damaged mitochondrial function, and neuro-inflammation, but also by oxidative stress [86]. Many studies have described molecules that are able to minimise the effects of Reactive Oxygen Species (ROS), and several studies are being undertaken to identify the availability of natural compounds that are effective in the treatment of NDs also acting as antioxidants [87,88]. As such, *Citrus* EOs have gained notable insight for this purpose. The EOs from *C.* × *clementina* leaves from Rosarno (Calabria, Southern Italy) exhibited promising activity as potential inhibitor of lipids peroxidation, with IC_50_ values of 4.76 and 15.07 μg/mL after 30 and 60 min of incubation in the β-carotene bleaching test [26]. This EO was active also as ABTS radical scavenger, with an IC_50_ value of 9.79 μg/mL. A lower activity level was found in the FRAP test.

Among the most abundant constituents of *C. medica* and *C.* × *clementina* EOs, myrcene showed in vivo promising antioxidant properties [72,89,90]. Ciftci et al. [89] analysed the effects of the treatment with the myrcene of female Sprague-Dawley rats exposed to 2,3,7,8-tetracholorodibenzo-*p*-dioxin. A decreased hepatic lipid peroxidation was observed. Increased levels of glutathione peroxidase, glutathione reductase and total glutathione in gastric tissue were observed after oral administration of β-myrcene (7.5 mg/kg bw; 55 μmol/kg bw) in male Wistar rats with ethanol-induced gastric ulcers [91]. In another work, glutathione peroxidase and superoxide dismutase activity was increased by myrcene [90], thereby preventing oxidative damage. Future studies aimed at investigating the antioxidant potentiality of β-myrcene require a comprehensive investigation into the recommended dosage of monoterpene in humans.

### 5.4. Enzymatic Inhibitory Activities

In the human body, enzymes are involved in many metabolic reactions, and the imbalance of their activity can lead to several diseases. Specific enzyme inhibitors have been developed and/or isolated from plants for the treatment of diseases that include hyperglycaemia, cancer, hypertension, neurodegenerative diseases such as Alzheimer’s disease, depression, and inflammatory diseases.

α-Amylase and α-glucosidase are enzymes involved in carbohydrate digestion and have been recognized as targets for the treatment of type 2 diabetes. In fact, the inhibition of these enzymes can significantly reduce the post-prandial increase of blood glucose and therefore can be an important strategy in the management of blood glucose level in type 2 diabetic and borderline patients. Inhibitors of these enzymes from plants offer an attractive strategy for the control of hyperglycaemia. In this context, the *Citrus* species has been extensively investigated.

EOs from *C.* × *clementina* leaves exhibited better activity in inhibiting α-amylase, with IC_50_ values in the range 135.51–148.64 μg/mL, with respect to α-glucosidase inhibition that had IC_50_ values in the range 151.27–282.65 μg/mL [26].

Among the most abundant constituents of *Citrus* EOs, 1,8-cineole (82.20%, 3.489 μL/mL) and R-(+)-limonene (70.25%, 2.633 μL/mL) exerted α-glucosidase-inhibitory activity with competitive and uncompetitive behaviour, respectively [92]. Previously, Basak and Canadan [93] reported that 1,8-cineole, at the concentration of 0.075 μL/mL, inhibited α-amylase with a percentage of 43.23%, whereas a value of 50% was found against α-glucosidase at the concentration of 1.118 μL/mL). No α-amylase-inhibitory activity was observed for sabinene and limonene at a concentration of 0.0670 mg/mL [94]. However, these compounds exerted antidiabetic potential by a different mechanism of action [95,96]. In general, researchers, based on their results, often hypothesize about the *Citrus* essential oil effects of synergism and antagonism of action to explain the enzymatic-inhibitory activity.

Tyrosinase is a multi-copper enzyme widely distributed in different organisms that plays an important role in the melanogenesis and enzymatic browning. Therefore, its inhibitors can be attractive in the medicinal and cosmetic industries as depigmentation agents, but also in the food and agriculture industries as anti-browning compounds. For this purpose, the search for natural, semi-synthetic and synthetic inhibitors is very active [97].

Leporini et al. [26] analysed several *C.* × *clementina* leaf extracts and EOs from three Southern Italy localities against tyrosinase and found weak activity for EOs, with IC_50_ values in the range 240.44–253.14 μg/mL. However, the other extracts obtained by Soxhlet apparatus, maceration, and ultrasound-assisted maceration also showed weak inhibitory activity, with IC_50_ values ranging from 150.52 to 280.33 μg/mL.

Thirteen *Citrus* EOs and their dominant constituents were previously investigated as tyrosinase-inhibitory agents [98]. Eureka lemon, Lisbon lemon, Keraji, and Kiyookadaidai were the most active in inhibiting the oxidation of l-dihydroxy phenylalanine (l-DOPA) by mushroom tyrosinase. Among volatile compounds, citral and myrcene revealed tyrosinase-inhibitory activity, with K_i_ values of 0.318 and 2.38 mM, respectively. The authors demonstrated that citral is a non-competitive inhibitor and myrcene is a competitive inhibitor. The controversial literature data against β-myrcene may be ascribed to the different substrates used for experimental tests. In fact, Capetti et al. [99] utilized L-tyrosine as a substrate, whereas L-DOPA was used by Matsuura et al. [98]. Based on the literature, it is possible to emphasize that β-myrcene may be more effective at inhibiting mushroom tyrosinase monophenolase activity than the diphenolase one. The activity of citral was confirmed by Capetti et al. [99], who found an IC_50_ value of 121.8 μg/mL. In the same work, an IC_50_ value of 13.3 μg/mL was registered for β-myrcene. Concentration-dependent inhibitory activity was also found for linalool and citronellol, with IC_50_ values of 730 and 825 μg/mL, respectively. However, this activity is negligible when compared to kojic acid [100].

### 5.5. Application in Foods

The increasing demand for high-quality and safe packaging materials has resulted in the longer shelf life of foods packed with eco-friendly materials. This needs natural base materials for packaging applications, along with ingredients that can extend the food shelf life. One such development in the field of active packaging is packaging that uses antimicrobial agents. Out of the many antimicrobials used, EOs are gaining importance due to their high activity. In this context, *Citrus* EOs have a key role and have been the object of different investigations. Bora et al. [101] recently reviewed the applications of *Citrus* EOs for food safety, preservation and packaging, with particular attention paid to the optimum dose and safe limits, the interaction effects with various food matrices and packaging materials, and possible allergic reactions. In this work, authors reported data about *C. limon*, *C. aurantifolia*, *C. sinensis*, *C. paradisi*, *C. aurantium*, *C.* × *paradisi*, *C. deliciosa*, and *C. reticulata*. The addition of some *Citrus* EOs, such as orange, lemon and mandarin, into gelatin films has provided promising antimicrobial activity in several studies [102,103,104,105].

Alparslan and Baygar [103] showed that chitosan films containing 2% orange peel EO were able to inhibit lipid oxidation and microbial growth, thereby extending the shelf-life of shrimp by nearly 8 days compared to the non-coated shrimp. Previously, chitosan film with lemon EO showed antimicrobial properties and enhanced the post-harvest quality of strawberries [104]. In another study, the antimicrobial activity of eight EOs extracted from the fruit peel of orange, lemon, and mandarin was evaluated against 76 strains of *Listeria monocytogenes* [105]. EOs showed the highest antibacterial activity when incorporated into methylcellulose- or chitosan-based biodegradable films.

Some studies also reported the potentiality of *C. medica*. No works are present in the literature on *C.* × *clementina*. The antibacterial effects of the combination of *Citrus* fruits, including *C. medica* EO, and some components (carvacrol and thymol) against non-acid-adapted/acid-adapted *E. coli* O157:H7, *Salmonella typhimurium*, and *Listeria monocytogenes* were examined [106]. Individual treatment with a mixture of 100% *C. medica*, 100% *C. limon* and 100% *C. macrocarpa* or carvacrol + thymol (from 1.0 to 2.0 mM) did not lead to a significant reduction in the population of the tested non-acid-adapted bacterial strains (initial population: 7.1–7.5 log CFU/mL), except for the treatment with 2.0 mM carvacrol + thymol, which reduced the growth of *S. typhimurium* (<2.2 log CFU/mL). From a sensorial point of view, the addition of carvacrol + thymol does not alter the consumer’s perceptions at concentrations up to 1.5 mM. More recently, Mitropoulou et al. [107] proposed the addition of *C. medica* peel EO to low alcohol wines (~6% vol) in a concentration of 0.010% *v*/*v* to act as bio-preservatives against spoilage bacteria and yeast. The EOs exert antimicrobial growth, with MIC values ranging from 530 to 2506 mg/L for *Z. bailii* and *H. uvarum*, and *P. pentosaceus*, respectively. The supplementation with *C. medica* alone or in combination with *C. zeylanicum* EO considerably reduced spoilage and caused microbial growth delay.

Khorsandi et al. [108] investigated the effect of *C. medica* EO lactic acid bacteria (LAB) (*Lactobacillus curvatus*, *Weissella viridescens*, *Leuconostoc mesenteroides*, *Enterococcus faecium*, *Lactobacillus reuteri*, *Lactobacillus dextrinicus*, *Lactobacillus sakei*, and *Pediococcus dextrinicus*) isolated from vacuum-packed cooked cured sausages. MIC values ranging from 7.33 to 9.33 mL/mL were found for *L. curvatus* and *L. dextrinicus*, respectively, whereas MBC ranged from 12 to 15 mL/mL for *L. curvatus*, *E. faecium* and *L. reuteri*, respectively.

This study demonstrated that sausage-isolated LAB are very sensitive to the action of the EO (inhibition zone diameter from 14 to 22 mm).

Considering the importance of fats in diets and the adverse effects of synthetic antioxidant agents, the antioxidant potential of *C. medica* peel EO together with other extracts (methanol, ethanol, and aqueous) was assessed on the thermal stability of sunflower oil [109]. *C. medica* peel showed to be a promising substitute of synthetic antioxidants.

Among *Citrus* EO dominant constituents, d-limonene was the most important for its applications in the food industry. This monoterpene was included in the list of compounds generally recognized as safe (GRAS) [110], and its use as flavouring agent in a variety of food products is regulated by sections 201(s) and 409 of the Federal Food, Drug, and Cosmetic Act (the Act) and implementing regulations by the US Food and Drug Administration. D-limonene is used in bakery products, juices, ice cream, etc., due the pleasant lemon-like odour. Moreover, it can be used as a safe antimicrobial agent [111].

Additionally, the monoterpene was characterized by its antioxidant potential, thus avoiding the post-harvest decay for processing and storage/packaging processes and extending the shelf-life of food products [112]. However, d-limonene was characterized by a relatively poor stability, especially in aqueous solutions. Moreover, it is volatile in the presence of air, moisture, and high temperatures. This monoterpene undergoes oxidative processes very easily, thus generating off-flavours [113]. Therefore, finding solutions that firstly protect this monoterpene against oxidation and secondly achieve its release in the required time and in a controlled manner is very important and is a turning point in the production and application of d-limonene in the food industry.

Akhavan-Mahdavi et al. [111] reviewed several methods to protect d-limonene and enhance its stability by the application of micelles, polymeric nanoparticles, nanoliposomes, solid lipid nanoparticles, nanostructured lipid carriers, nanosuspensions, and nanoemulsions For example, Dhital et al. [114] created a d-limonene liposome to be used in a coating formulation with alginate solution to improve the shelf life of strawberries for 14 days of storage at 4 °C. Samples coated with d-limonene liposomes are characterized by a lower respiration rate and higher anthocyanin content than the untreated samples. A similar situation was also observed when liposomal-encapsulated d-limonene was used in a coating formulation to protect blueberries against microbial deterioration for >9 weeks at 4 °C [115]. The nanoemulsion formulated with ε-polylysine and d-limonene is characterized by an improved antimicrobial activity against food pathogens versus the control sample [116]. Polymeric nanoparticles formulated with methyl methacrylate and triethylene glycol dimethacrylate copolymers were used as matrix-carriers for hosting d-limonene.

These nanoparticles are characterized by an improved antimicrobial activity in comparison to the d-limonene alone. These nanoparticles should be used in food packaging applications [117]. The stability of flavours in different food matrices is an important issue during thermal processing. To minimize the loss of aroma in rock candy, Lotfabadi et al. [118] proposed the encapsulation of d-limonene using gum Arabic and maltodextrin.

Authors evidenced that the presence of sucrose and citric acid increases d-limonene release because of a salting-out effect, and it also significantly affects its perception. Even though R-(+)-limonene is used as a common flavour additive in a variety of food products, it can induce toxic effects, especially in rats. For this reason, it can be categorized as a low-toxic additive, however, additional studies are required about the toxicity of d-limonene metabolites and their safe scientific limits [119].

## 6. Conclusions and Future Perspectives

The great interest in *Citrus* EOs mainly originates from two factors. First, there is a great availability of EOs, also as by-products, because *Citrus* is the largest fruit crop. Secondly, the biological properties and fragrance of *Citrus* EOs make them precious constituents of perfumes and cosmetics and promising substitutes to the currently used antimicrobial agents. *C. medica* and *C.* × *clementina* EOs have been demonstrated to possess these characteristics. Moreover, these EOs are eco-friendly, economic, and a natural alternative to synthetic preservatives for the food industry. However, the use of these EOs is limited due to their hydrophobicity, high volatility, and instability [120,121]. Therefore, today, there are many studies that have investigated the possibility of using innovative formulations for the encapsulation of *Citrus* EOs, not only to preserve their flavouring characteristics but also to formulate innovative packaging systems (edible films, nanoemulsion coatings, and microencapsulated polymers). Furthermore, *Citrus* EOs are characterized by their antimicrobial effect, and for this reason they will be useful for the post-harvest disease control of fruits and vegetables.

However, some fundamental aspects of their use require clarification, in particular their optimal dose and safety limits as well as their interactions with some food matrices and packaging materials. These aspects require further extensive research to ensure the safety of these natural products.

## Figures and Tables

**Table 1:** The main constituents (%) of *C. medica* cv. Diamante peel EO from Italy.

Compound	[27] *	[28] *	[29] ^#^
Thujene	1.2	0.9	0.28–0.59
α-Pinene	2.5	2.1	0.69–1.47
Camphene	0.9	tr	tr–0.01
Sabinene	0.4	0.2	0.14–0.24
β-Pinene	2.6	1.9	0.93–1.39
Myrcene	2.1	1.6	1.13–1.47
α-Phellandrene	0.2	0.1	0.04–0.05
α-Terpinene	1.2	0.5	0.35–0.41
*p*-Cymene	tr	0.5	-
Limonene	35.4	60.8	51.27–60.56
(Z)-β-Ocimene	-	1.4	0.75–1.19
(E)-β-Ocimene	2.4	1.9	1.10–1.74
γ-Terpinene	24.5	23.4	20.89–24.40
Terpinolene	1.5	1.0	0.87–1.08
Linalool	0.6	0.1	0.17–0.37
*n*-Nonanal	0.2	0.1	0.04–0.09
Citronellal	0.3	0.1	0.04–0.08
Terpinen-4-ol	1.5	0.1	0.04–0.10
α-Terpineol	1.1	0.2	0.21–0.61
*n*-Decanal	0.1	tr	0.04–0.07
Nerol	1.0	0.1	-
Neral	4.4	0.8	1.12–3.77
Geraniol	0.3	0.1	0.01–0.22
Perilla aldehyde	0.2	-	0.01–0.02
Geranial	5.5	1.0	1.80–6.26
Citronellyl acetate	0.2	tr	0.01–0.04
Neryl acetate	0.5	0.2	0.11–0.20
Geranyl acetate	0.4	0.2	0.19–0.41
β-Elemene	0.1	tr	0.01–0.02
β-Cubebene	0.1	-	-
β-Caryophyllene	0.3	0.1	0.10–0.22
*trans*-α-Bergamotene	0.1	0.2	0.28–0.48
*trans*-β-Farnesene	0.5	tr	tr
α-Humulene	0.2	tr	0.03–0.06
β-Bisabolene	1.2	0.3	0.40–0.67
Tetradecanal	tr	-	0.01–0.02
γ-Cadinene	tr	tr	-
δ-Cadinene	0.2	-	-
Germacrene B	-	tr	-
(E)-Nerolidol	-	tr	-
Spathulenol	-	tr	-
β-Bisabolol	0.4	tr	-
Citropten	0.9	-	-

* The hydrodistillation method of fresh peel was reported. ^#^ Value for the range of EOs obtained by direct aspiration, manual squeezing extraction and manual abrasion extraction from small green, big green and yellow fruits. tr: traces (<0.1%).

**Table 2:** The main volatile compounds (%) identified in ì *C. clementina* peel.

Compound	Peel				
	[38] *	[39] *	[40]	[41] **	[42]
α-Pinene	1.27	0.60	0.47	1.10–3.13	0.43
Camphene	-	-	0.38	-	-
Sabinene	0.83	0.20	-	3.56–9.10	0.15
β-Pinene	-	-	1.83	-	-
*n*-Octanal	0.44	0.20	0.37	-	0.13
Myrcene	4.64	0.95	-	3.56–9.10	1.82
δ-3-Carene	0.21	-	-	-	0.05
α-Phellandrene	0.05	-	-	-	-
δ-3-Carene	0.06	0.05	-	0.22–0.36	-
α-Terpinene	0.05	-	-	-	-
*p*-Cymene	0.05	-	-	-	-
Limonene	88.12	95.03	94.77	61.31–83.09	95.46
(E)-β-Ocimene	-	-	-	3.31	-
γ-Terpinene	0.05	-	-	0.32–0.33	-
Terpinolene	0.05	-	-	0.30	-
Linalool	1.02	0.30	0.82	3.39–6.64	0.53
Citronellal	0.40–0.75	0.08	0.07	-	0.05
Terpinen-4-ol	-	-	0.05	0.20–0.88	-
α-Terpineol	0.15	0.05	0.12	1.55	0.07
*n*-Decanal	0.71	0.18	0.34	0.47–1.55	0.27
Nerol	-	0.05	-	-	-
Geranial	-	-	0.06	-	-
Neryl acetate	0.06	-	-	-	-
Geranyl acetate	0.06	-	-	-	-
β-Copaene	0.10	-	-	-	-
β-Elemene	0.05	-	-	-	-
β-Cubebene	0.10	-	-	-	-
Dodecanal	0.18	-	0.06	-	0.05
β-Caryophyllene	0.05	0.06	-	-	-
β-Bisabolene	-	0.07	-	-	-
γ-Muurolene	-	0.05	-	-	-
δ-Cadinene	0.06	0.05	-	0.22–0.36	-
(E)-Nerolidol	0.05	-	-	-	-
β-Sinensal	0.10	-	-	0.22–0.31	-
α-Sinensal	0.30	0.07	0.11	0.37–0.70	0.07

* The best extraction method was reported. ** Value for the range of the following three sites of collection: Cetraro, Rosarno and Corigliano Calabro. Note: Values below 0.05% are not considered.

**Table 3:** The main volatile compounds (%) identified in *C. clementina* leaves.

Compound	Leaves	
	[39] *	[26] **
Thujene	0.15	0.51–1.43
α-Pinene	0.66	4.73–5.0
Sabinene	19.52	22.59–23.32
β-Pinene	1.12	-
Myrcene	1.75	4.22–4.45
δ-3-Carene	-	6.33–7.06
α-Phellandrene	0.08	1.37–1.59
δ-3-Carene	0.07	0.19–0.28
α-Terpinene	0.41	2.08–2.64
Limonene	1.95	5.88–6.62
(Z)-β-Ocimene	0.29	-
(E)-β-Ocimene	5.0	6.52–7.16
γ-Terpinene	0.97	3.01–3.72
Terpinolene	-	2.89–3.14
Linalool	7.51	10.41–16.83
Citronellal	-	2.87
Terpinen-4-ol	0.06	2.43–4.54
α-Terpineol	-	0.38–1.40
*n*-Decanal	-	0.14–0.17
Nerol	-	0.81–1.27
Neral	-	0.14–0.16
Geranial	-	0.12–0.20
Citronellyl acetate	0.32	-
Neryl acetate	-	0.13
Geranyl acetate	18.04	0.20–0.64
β-Elemene	7.49	0.24
β-Cubebene	-	0.24
β-Caryophyllene	0.13	0.69–1.92
*trans*-β-Farnesene	-	0.34–0.75
α-Humulene	-	0.11–0.22
γ-Elemene	2.30	-
β-Bisabolene	0.13	-
α-Muurolene	5.32	-
γ-Muurolene	0.41	-
α-Selinene	3.06	-
δ-Cadinene	0.07	0.19–0.28
Germacrene B	-	0.31–1.04
(E)-Nerolidol	0.52	0.20–0.35
Spathulenol	0.06	-
γ-Eudesmol	0.49	-
α-Muurolol	1.31	-
β-Sinensal	-	3.14–4.77
α-Sinensal	-	1.46–2.68

* The best extraction method was reported. ** Value for the range of the following three sites of collection: Cetraro, Rosarno and Corigliano Calabro. Note: Values below 0.05% are not considered.

**Table 4:** The main volatile compounds (%) identified in *C. clementina* flowers.

Compound	Flowers
	[43]
α-Pinene	0.8–1.2
Sabinene	34.8–47.9
β-Pinene	1.8–2.2
Myrcene	2.4–3.2
δ-3-Carene	0.2–0.4
*p*-Cymene	1.7–2.4
Limonene	6.2–9.6
(Z)-β-Ocimene	0.1
(E)-β-Ocimene	0.5–1.4
γ-Terpinene	0.1–0.6
Terpinolene	0.1–0.2
Linalool	16.8–28.9
Terpinen-4-ol	3.0–3.9
α-Terpineol	0.5–0.9
β-Elemene	0.2–0.7
β-Caryophyllene	0.2–1.3
β-Caryophyllene oxide	0.3–0.9
(E)-Nerolidol	4.2–7.1

## Data Availability

Not applicable.
